# H3 K36 Methylation Helps Determine the Timing of Cdc45 Association with Replication Origins

**DOI:** 10.1371/journal.pone.0005882

**Published:** 2009-06-12

**Authors:** Fiona Pryde, Devanshi Jain, Alastair Kerr, Rebecca Curley, Francesca Romana Mariotti, Maria Vogelauer

**Affiliations:** Wellcome Trust Centre for Cell Biology, Institute of Cell Biology, University of Edinburgh, Edinburgh, United Kingdom; Duke University, United States of America

## Abstract

**Background:**

Replication origins fire at different times during S-phase. Such timing is determined by the chromosomal context, which includes the activity of nearby genes, telomeric position effects and chromatin structure, such as the acetylation state of the surrounding chromatin. Activation of replication origins involves the conversion of a pre-replicative complex to a replicative complex. A pivotal step during this conversion is the binding of the replication factor Cdc45, which associates with replication origins at approximately their time of activation in a manner partially controlled by histone acetylation.

**Methodology/Principal Findings:**

Here we identify histone H3 K36 methylation (H3 K36me) by Set2 as a novel regulator of the time of Cdc45 association with replication origins. Deletion of *SET2* abolishes all forms of H3 K36 methylation. This causes a delay in Cdc45 binding to origins and renders the dynamics of this interaction insensitive to the state of histone acetylation of the surrounding chromosomal region. Furthermore, a decrease in H3 K36me3 and a concomitant increase in H3 K36me1 around the time of Cdc45 binding to replication origins suggests opposing functions for these two methylation states. Indeed, we find K36me3 depleted from early firing origins when compared to late origins genomewide, supporting a delaying effect of this histone modification for the association of replication factors with origins.

**Conclusions/Significance:**

We propose a model in which K36me1 together with histone acetylation advance, while K36me3 and histone deacetylation delay, the time of Cdc45 association with replication origins. The involvement of the transcriptionally induced H3 K36 methylation mark in regulating the timing of Cdc45 binding to replication origins provides a novel means of how gene expression may affect origin dynamics during S-phase.

## Introduction

DNA replication of eukaryotic chromosomes starts at multiple loci called replication origins. A prereplicative complex (preRC) forms at these loci at the end of mitosis/early G1. This preRC remains inactive until the beginning of S-phase, when cyclin- and DBF4-dependent kinases (CDK and DDK, respectively) are activated. Their signal leads to a hierarchical association of replication factors at origins, and initiation of DNA synthesis [1]. One such replication factor, Cdc45, has been shown to associate with origins approximately at their time of activation [2]–[5].

Only a subset of replication origins is activated at any given time during S-phase, likely reflecting differences between replication origins in their efficiency of activation [6]–[10]. Differential timing in origin firing determines the number and distribution of replication forks along chromosomes and has important implications for genome stability. In fact, activation of late origins is inhibited upon DNA damage or replication stress [11]–[15]. Timing of replication origin firing is partly controlled by S-phase cyclins and DNA checkpoint kinases. In *Saccharomyces cerevisiae* the deletion of one of the S-phase cyclins, Clb5, causes a strong delay of late replication origins [16]. This delay results in inactivity of most late origins on the chromosome, as they are inactivated by the passing replication fork before they can fire. In contrast, inhibition of S-phase checkpoint kinases advances origin firing in both yeast and human cells [15], [17]. These findings support a model in which replication timing is the result of competing signals, which may determine the availability of replication factors to activate origins.

Replication factors, such as Cdc45, need to interact with replication origins embedded in their chromosomal context. It is therefore not surprising that the time of firing does not depend on the origin itself but on its chromosomal environment [18], [19]. This has been demonstrated in *Saccharomyces cerevisiae*, where origins consist of DNA segments of ∼200 bp, also named Autonomously Replicating Sequences (ARS), as they confer to episomal plasmids the ability to replicate. Transfer of an early firing origin to a late replicating region results in its late activation [19]. Moreover, late ARSs maintain their late timing on a plasmid only when transferred with several kilobases of their surrounding chromosomal sequence [19], [20]. Determinants of replication timing must therefore be inherent to the chromosomal context, albeit their precise nature remains to be uncovered.

A correlation between replication timing and transcriptional activity of proximal genes has been observed in many organisms, suggesting a connection between these nuclear functions. High resolution replication profiles reveal an overall positive correlation between gene expression and timing of replication in both human and *Drosophila melanogaster* [8], [9], [21]. However, several instances have been reported in which transcription by RNA polymerase II (RNA pol II) inactivates DNA replication origins. In *S. cerevisiae* the activity of a plasmid borne ARS is inhibited by transcription induced from an adjacent promoter [22]. Moreover ARS605, located within the open reading frame of a meiosis specific gene, is active when transcription is repressed in mitosis, but becomes inactivated upon transcriptional induction of this gene during meiosis [23]. Similarly, replication origins within the mammalian HoxB domain are silenced upon transcriptional activation of the locus [24]. Therefore, while proximity to transcribed genes may confer early activation timing to origins, the location within actually transcribed regions may inhibit their activation.

Similar to their regulatory role in transcription, histone modifications could regulate the access of replication factors to replication origins and therefore determine the time of origin activation. This has been proposed for histone acetylation. Inhibitors of histone deacetylases cause advanced replication timing, and late replicating chromosomal regions colocalize with hypoacetylated chromatin [25], [26]. Studies in *S.cerevisiae* showed that increasing global histone acetylation by deletion of the Rpd3 histone deacetylase results in earlier association of Cdc45 with late origins and advanced time of activation [2], [4]. Moreover, recruitment of a histone acetyltransferase (HAT) to a single late origin advances Cdc45 binding in yeast and time of firing in both human and yeast [4], [27]. Nonetheless, a genome wide correlation between histone acetylation and the time of origin firing has not been detected [28]. It is therefore unlikely that this modification is the sole determinant of replication timing.

The importance of multiple histone modifications in regulating transcription is well established. Furthermore, transcription itself leads to changes in the modification pattern of the underlying chromatin [29]. For instance, the Set2 histone methyltransferase binds directly to the phosphorylated C-terminal tail domain (CTD) of elongating RNA pol II [30]–[35]. This results in methylation of histone H3 lysine 36 over transcribed genes, which causes the recruitment of the small Rpd3 complex (Rpd3(S)), deacetylation of histones and repression of spurious transcription initiation [36]–[38]. H3 K36 can be mono-, di- and trimethylated (H3 K36me1, -me2 and -me3). While in yeast Set2 is responsible for all three states of K36 methylation (K36me), separate enzymes have evolved in higher eukaryotes to provide either K36me2 or K36me3 [39]. Recent studies indicate that the different methyl states of H3 K36 are functionally distinct. In *D.melanogaster* H3 K36me2 and -me3 have been shown to have opposite effects on H4 K16 acetylation [39] while in *Arabidopsis thaliana* K36me2 and -me3, but not -me1, are required for transcription of genes regulating flowering time [40]. In *S. cerevisiae* K36me3, but not -me2, is dependent on phosphorylation of the C-terminal domain of RNA pol II by Ctk1 and correlates with transcription [41]. The number of methylation moieties attached to K36 therefore profoundly affects the function of this histone residue.

Histone modifications often provide the binding substrate for histone-binding proteins. K36me3 has been shown to be directly bound by three different proteins: Ecm5, Eaf3 and Nto1. Ecm5 is a protein of unkown function, but the presence of a PHD-finger and Jumonji-C domain strongly suggest a role in either binding or regulating histone modifications [42]. Eaf3 is a subunit of both the small Rpd3 histone deacetylase complex and the NuA4 histone acetyltransferase complex, while Nto1 is part of the NuA3 histone acetyltransferase [36]–[38], [43], [44]. Together they provide a direct link between H3 K36me3 and the regulation of histone acetylation.

We show here that methylation of H3 K36 is involved in regulating the kinetics of Cdc45 association with replication origins. Its binding to origins is delayed in the absence of *SET2* and cannot be advanced by increasing histone acetylation in this genetic background. Furthermore, our data are consistent with K36me1 and -me3 having opposing functions in DNA replication initiation. K36me1 increases at replication origins upon binding of Cdc45, suggesting a positive function for K36me1 during initiation of DNA replication. On the contrary, early origins are depleted of K36me3 and this modification decreases around the time of Cdc45 binding to origins, pointing to a negative role of this modification. This is further supported by a shortened S-phase in the absence of the K36me3-binding proteins Eaf3 and Nto1. We propose that a combination of multiple histone modifications regulates the timing of replication origin firing.

## Results

### H3 K36me by Set2 is necessary for accelerated S-phase progression in the absence of *RPD3*

**Figure 1:**
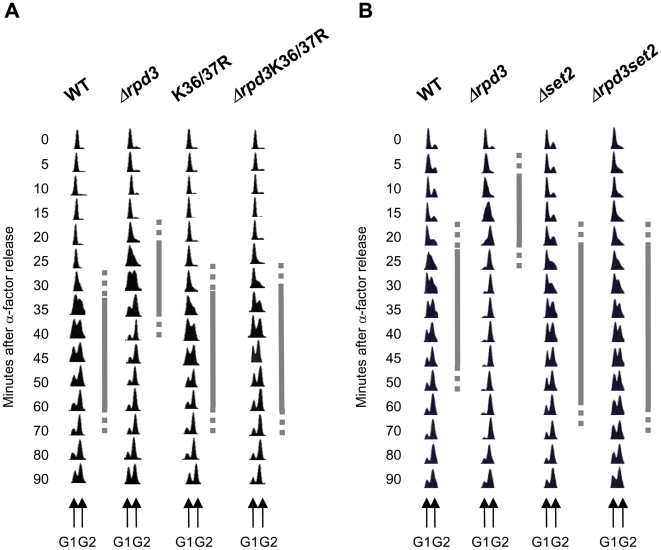
H3 K36me by Set2 is necessary for accelerated S-phase progression in *Δrpd3* cells. (A) Exponentially growing cells of strains MVY17 (WT), MVY31 (Δ*rpd3*), MVY37 (K36/37R) and MVY34 (Δ*rpd3*K36/37R) were arrested in G1 with α-factor and released into S-phase at 30°C. Samples were taken at indicated times and processed for FACS analysis. G1 and G2 DNA content are indicated at the bottom of the figure. Grey bars indicate the estimated length of S-phase. (B) Same as (A) but with strains MMY001 (WT), MMY002 (Δ*rpd3*), MVY42 (Δ*set2*) and MVY43 (Δ*rpd3*Δ*set2*).

Deletion of the Rpd3 histone deacetylase in *S.cerevisiae* leads to earlier Cdc45 binding at late origins and concomitant advancement in time of activation, resulting in a more rapid S-phase progression [2], [4]. To better understand the molecular mechanisms involved in this process we asked which histone lysine residues were necessary for the accelerated S-phase in *Δrpd3* cells. We therefore deleted *RPD3* in strains carrying different combinations of histone lysine (K) to arginine (R) substitutions and determined which mutations would revert the more rapid S-phase. α-factor arrested cells (G1) were released into S-phase at 30°C and their DNA content at indicated times determined by FACS (Fig. 1A and data not shown). Although this assay is not a precise measure for the length of S-phase, it is sensitive enough to determine major differences between strains. It should also be noted that G1 release and S-phase progression can differ between experiments. Therefore, all kinetics presented within one figure-panel show results from strains grown and processed in parallel. A preliminary experiment using a H4 K5/8/12/16R mutant did not alter the more rapid S-phase when *RPD*3 is deleted. A H3 K4/9/14/18/23/27R mutant caused a prolonged cell-cycle in the absence of *RPD3*, which rendered the analysis of difficult interpretation, while the H3 K27R mutant still allowed for a more rapid S-phase upon deletion of *RPD3* (data not shown). However, when H3 K36/37R was used as genetic background, deletion of *RPD3* no longer led to S-phase shortening (Fig. 1A). DNA synthesis in the WT strain occurred between ∼30 and 70 min after release from G1. As expected, both entry into and progression through S-phase were accelerated in the *Δrpd3* strain with DNA synthesis occurring between ∼20 and 40 min. This is not due to a general shortening of the cell cycle. In fact, both WT and *Δrpd3* cells enter mitosis at ∼90 min, as indicated by the reappearance of the G1 peak. The K36/37R mutant progressed through S-phase with kinetics similar to the WT. Strikingly, when *RPD3* was deleted in this histone mutant no shortening of S-phase was observed. Therefore, H3 K36 and/or K37 are necessary for more rapid DNA replication in the absence of histone deacetylation by Rpd3.

To test if methylation of H3 K36 by Set2 was necessary for a more rapid S-phase in the absence of Rpd3, *SET2* was deleted in a WT and its isogenic *Δrpd3* strain. S-phase progression was monitored as described above (Fig. 1B). As seen before cells lacking *RPD3* showed a reduction in the time of S-phase progression compared to WT. *Δset2* cells had a slightly longer S-phase than the WT. We consistently observed a certain percentage of *Δrpd3Δset2* cells remaining in G1 without entering the cell cycle. Such a sub-population was observed in all six repeats of this experiment and may reflect a secondary effect of the mutation. The remaining *Δrpd3Δset2* cells however, replicated with similar times as the *Δset2* mutant, again abolishing the more rapid S-phase in absence of *RPD3*. This parallels the behaviour of the K36/K37R mutation and argues that methylation of K36 by Set2 is necessary for the accelerated S-phase observed in *Δrpd3* cells.

### Set2 is necessary to advance Cdc45 association with origins in the absence of *RPD3*

**Figure 2:**
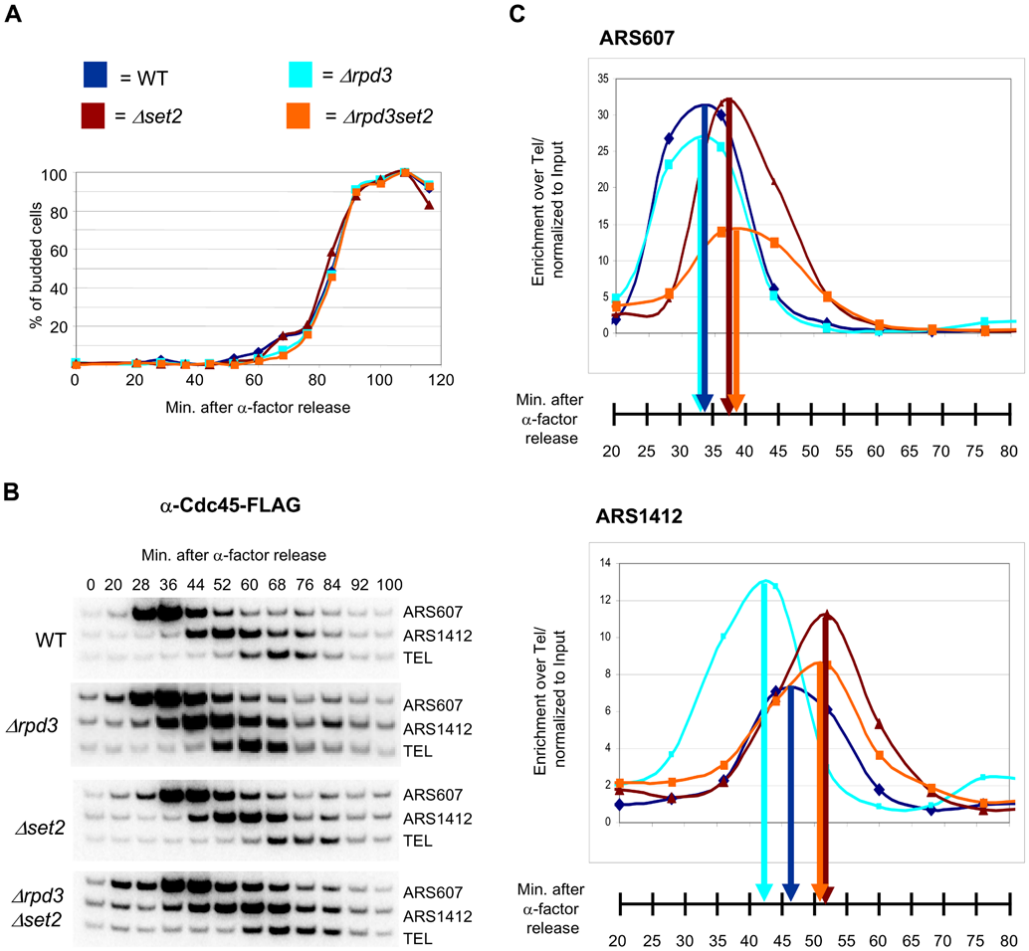
Set2p is necessary for advanced association of Cdc45 with origins in WT and *Δrpd3* cells. Strains MMY033 (WT), MVY51 (Δ*rpd3*), MVY57 (Δ*set2*) and MVY58 (Δ*rpd3*Δ*set2*) were arrested in G1 with α-factor, released at 24°C into S-phase and samples were taken at indicated times. (A) Cell budding was assessed by microscopy. (B) ChIP of Cdc45-3FLAG was performed with an α-FLAG antibody and analysed by semiquantitative PCR using primers specific for ARS607, ARS1412 and a telomeric loading control (TEL). (C) Graphical representation of Cdc45-3FLAG ChIP showing the relative intensity of ARS-specific fragments after normalization to the telomeric loading control fragment and the input DNA. The time of maximal intensity is indicated for each strain.

Deletion of *RPD3* had been shown to accelerate S-phase progression by advancing Cdc45 binding and activation of late replication origins [2], [4]. To test whether H3 K36me is necessary for this advanced binding of Cdc45, we determined the time of Cdc45 association with eight origins, comparing WT, *Δrpd3*, *Δset2* and *Δrpd3Δset2* strains. WT, *Δrpd3*, *Δset2* and *Δset2Δrpd3* strains expressing a FLAG-tagged version of Cdc45 were arrested in G1, released into S-phase at 24°C to allow for better resolution and samples taken at indicated times. ChIP was performed using αFLAG-antibody and the resulting DNA analysed by semiquantitative PCR (Fig. 2B). Linearity of the reaction was tested by amplification of increasing amounts of ChIP- and Input-DNA (Fig. S1). A very late replicating telomeric sequence was used as internal control. Although this sequence associates with Cdc45p as the replication fork moves across, it does so later and was therefore considered to be the best choice in order to control for loading differences. FACS analysis determined S-phase progression (Fig. S2), while cell-budding demonstrated a synchronous progression through the cell cycle for all four strains (Fig. 2A). This analysis was repeated twice, with comparable results. Moreover, two additional repeats using the *Δset2* and *Δrpd3set2* strains further confirmed the results described below.

Cdc45 association with the early ARS607 peaked between 28 and 36 min in both the WT and *Δrpd3* strains, but was delayed in *Δset2* and *Δrpd3Δset2* strains (between 36 and 44 min) (Fig. 2B and 2C). At the late firing ARS1412 binding of Cdc45 peaked between 44 and 52 min in the WT and peaked earlier in the *Δrpd3* strain by approximately one time-point. This confirms the advanced activation of late origins previously observed in *Δrpd3* cells [2], [4]. Association of Cdc45 at ARS1412 was again delayed in the *Δset2* strain and, most importantly, was not advanced upon deletion of *RPD3* (*Δrpd3Δset2*). Similar results were obtained at six more ARSs, with the exception of ARS1524 where Cdc45 binding was unaffected in the *Δrpd3* strain (Fig. S3). These data show that deletion of *SET2* delays Cdc45 binding to replication origins and eliminates its earlier association with late origins in the absence of *RPD3*.

### *Δset2* does not decrease histone acetylation at origins

**Figure 3:**
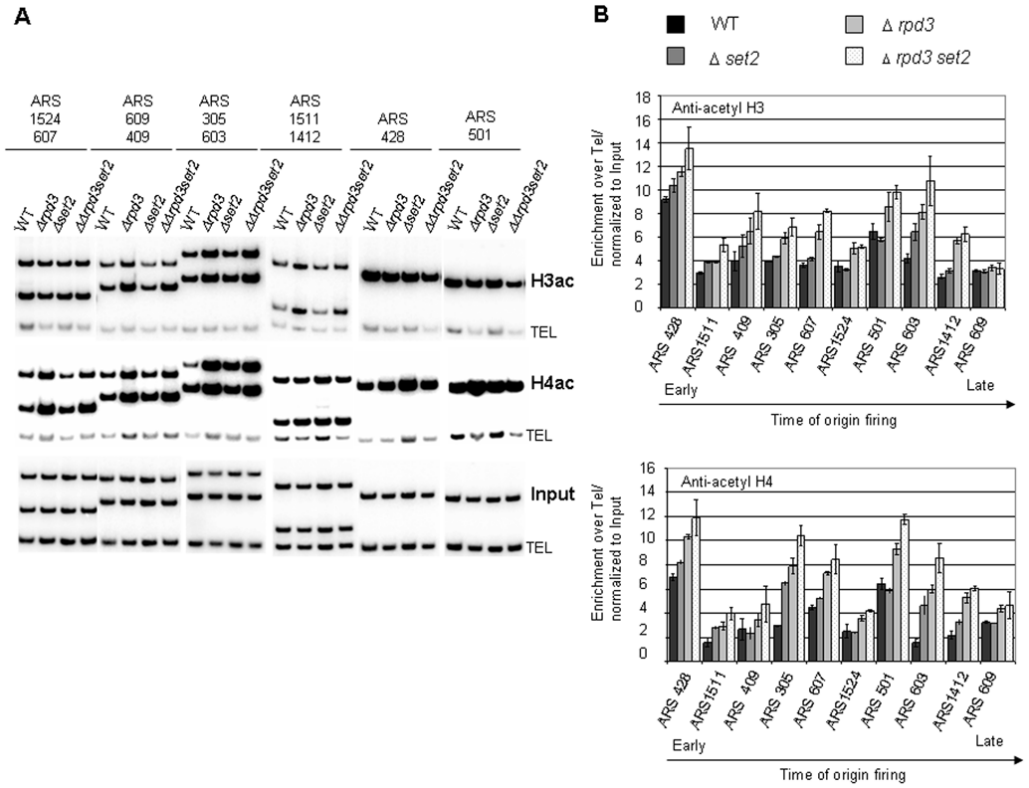
*Δset2* does not decrease histone acetylation at origins. ChIP of MMY033 (WT), MVY51 (Δ*rpd3*), MVY57 (Δ*set2*) and MVY58 (Δ*rpd3*Δ*set2*) with antibodies specific for pan-acetylated histone H3 and H4 was performed. (A) Representative autoradiographs of PCR products using primer pairs specific for the indicated ARS elements and a telomeric loading control (TEL). (B) Graphic representation: Analysis was by semiquantitative PCR using primer pairs specific for indicated ARS elements and a telomeric loading control. The relative intensity of ARS specific fragments after normalization to the loading control and the input is presented. Errorbars refer to the standard deviation of the results of three independent experiments.

To rule out the possibility that the *SET2* deletion reduces histone acetylation at origins and therefore indirectly affects the dynamics of Cdc45 binding we analysed the level of histone acetylation at ten origins in WT, *Δrpd3*, *Δset2* and *Δrpd3Δset2* strains. Antibodies against acetylated histones H3 or H4 were used for ChIP. Deletion of *SET2* alone led to a slight increase in H3 and H4 acetylation at most origins when compared to the WT and was further increased in the absence of *RPD3* (Fig. S4). This is expected, as H3 K36me3 is known to recruit the Rpd3S histone deacetylase complex. Two origins (ARS607 and ARS603) showed a 2–3 fold increase in H4 acetylation in Δ*set2* cells compared to the WT. Importantly, H3 and H4 acetylation levels in the *Δrpd3* mutant were not diminished upon deletion of *SET2* (Fig. 3 and Fig. S4). *Δrpd3Δset2* cells showed a slight overall increase (up to ∼1.5 fold) in histone acetylation when compared to the *Δrpd3* strain (Fig. 3B). We conclude that the absence of H3 K36me delays Cdc45 binding to origins even when the surrounding chromatin is hyperacetylated.

### *Δrpd3* does not significantly increase H3 K36me at replication origins

**Figure 4:**
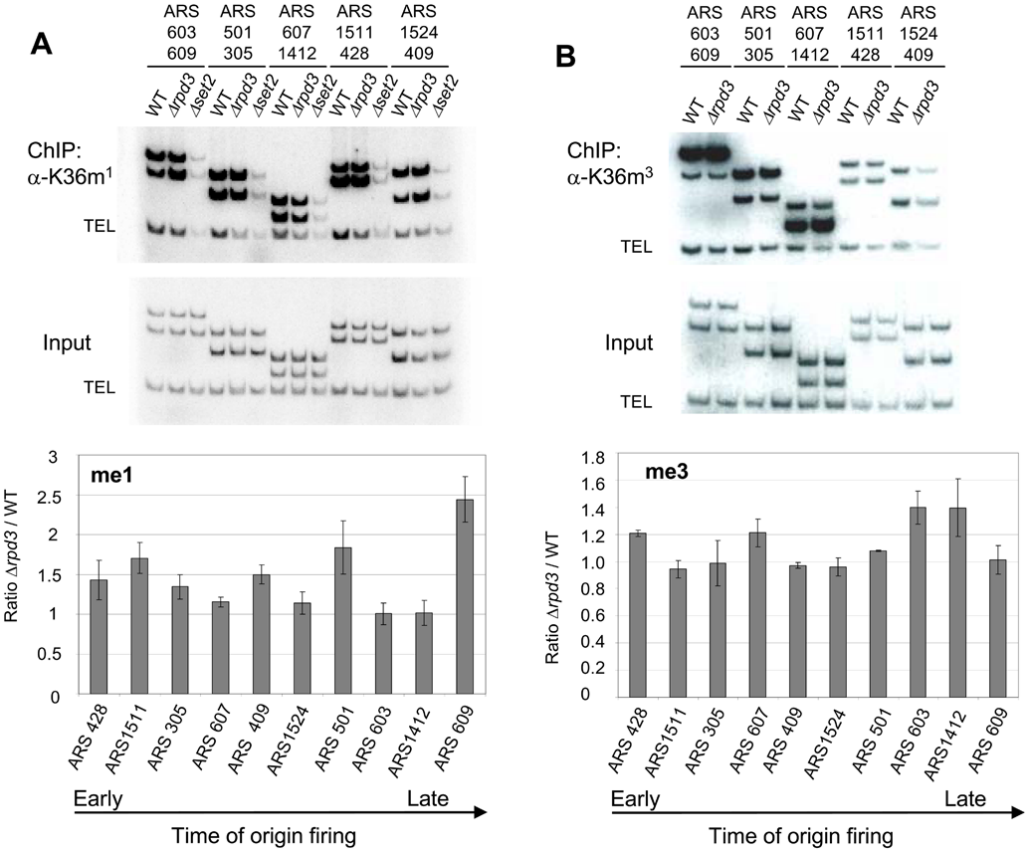
*Δrpd3* increases K36me at some but not all origins. ChIP of MMY033 (WT) and MVY51 (Δrpd3) with antibodies specific for K36me1 (A) and −me3 (B). ChIP of strain MVY57 (Δ*set2*) is included as a negative control in (A). Representative autoradiographs of PCR products using primers specific for the indicated ARS elements and a telomeric loading control (TEL). The graph represents the average ratio (Δ*rpd3*/WT) of three independent experiments after normalization to input DNA and loading control. Error-bars refer to the standard deviations thereof.

With similar reasoning it is possible that histone acetylation could lead to increased K36me at origins and indirectly determine their time of activation. We therefore determined levels of K36me1 and -me3 at the same ten ARSs by ChIP in WT and *Δrpd3* strains. Commercially available antibodies against K36me2 gave a very poor signal and were excluded from our analysis. Six out of ten origins show levels of K36me1 increased more than 1.3 fold in the *Δrpd3* strain when compared to the WT, with the highest increase at subtelomeric ARS609 (approximately 2.5 fold) (Fig. 4A). Although these data indicate a tendency for increased levels of K36me1 in the absence of *RPD3*, no increase of K36me1 was observed in the *Δrpd3* strain at late ARS1412 and ARS603, although Cdc45 binding is advanced in this genetic background [4] (Fig. 2 and Fig. S3). Moreover, there is no correlation between K36me1 and the timing of these origins (Fig. S5A and S5B). Levels of K36me3 were even less affected by the deletion of *RPD3.* Only four out of ten origins showed a 1.2–1.4 fold increase of this histone modification (Fig. 4B). Taken together, these results show that advanced Cdc45 binding to origins in the *Δrpd3* strain cannot simply be explained by increased levels of either K36me1 or -me3.

### Early origins are depleted in K36me3 compared to late firing origins

**Figure 5:**
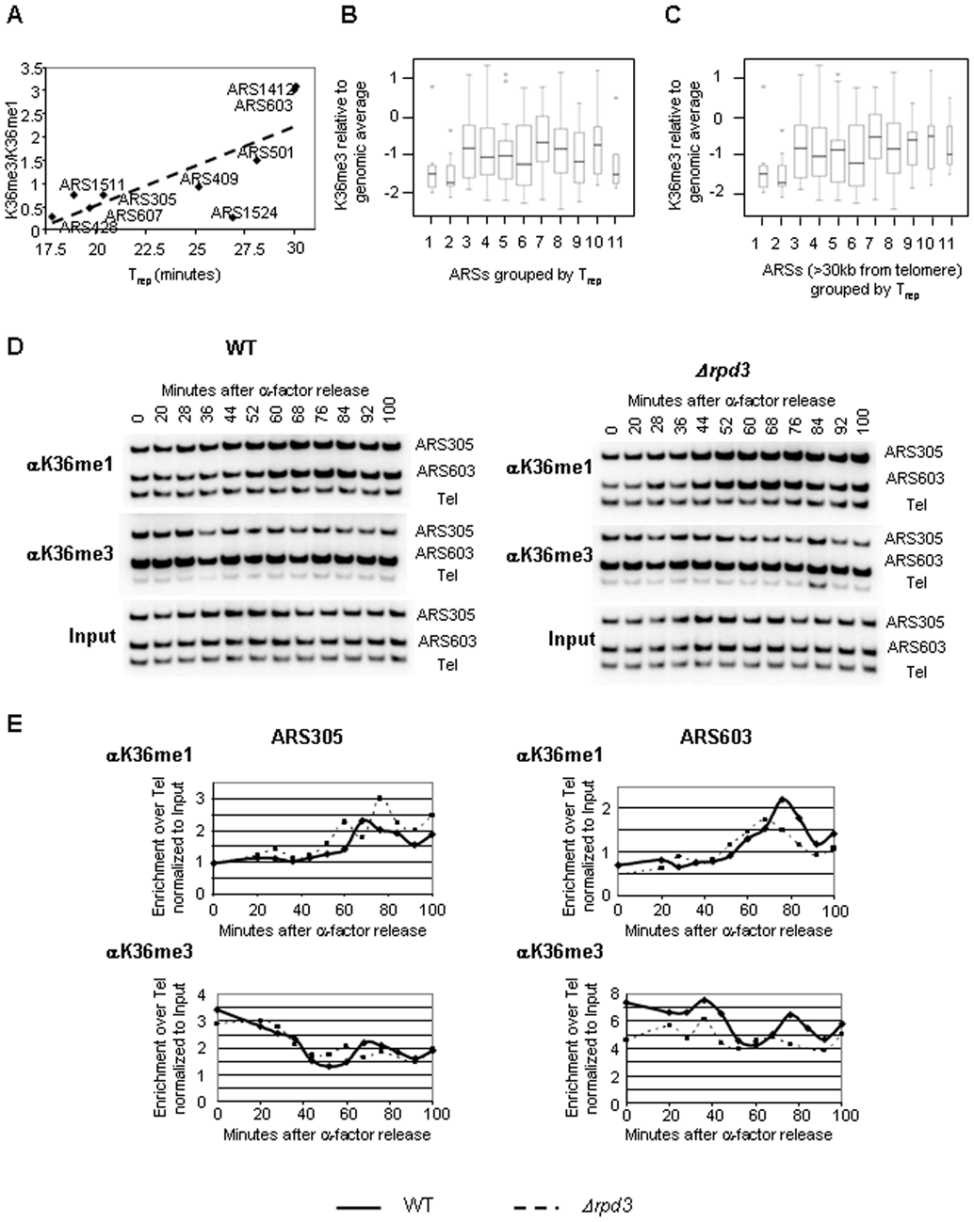
K36me1 and K36me3 show opposing behaviour at DNA replication origins. (A) The ratio of K36me3/K36me1 for indicated ARSs is plotted against their time of activation and a linear trend-line was drawn. (B) ARSs are grouped for their time of firing in three minutes time intervals relative to the firing of the earliest ARS [6]. ChIP on CHIP (Nimblegen) was used to determine genome-wide levels of H3 K36me3 and their distribution was determined for each group of ARSs and represented as box-plot. W- and P-values described in the text were calculated using the Wilcoxon rank sum test with continuity correction. (C) Same as in (B), but excluding all ARSs within 30 kb from chromosomal ends. (D) ChIP of MMY033 (WT) and MVY51 (Δ*rpd3*) with antibodies specific for K36me1 and −me3. Representative autoradiographs of PCR products using primers specific for the indicated ARS elements and a telomeric loading control (TEL). (E) Graphical representation of K36me1 or K36me3 ChIP showing the relative intensity of ARS-specific fragments after normalization to the loading control and the input. Complete lines indicate WT and broken lines indicate Δ*rpd3*.

High levels of K36me3 were observed only at origins to which Cdc45 binds at later timepoints (Fig. S5A and S5B, Fig. 2 and Fig. S3). This was surprising given the apparent positive role of Set2 in Cdc45 binding to origins. As deletion of *SET2* abolishes both K36me1 and –me3, we considered the possibility that these two modifications may have opposite effects. In WT yeast cells, the time of activation of any given origin correlates with the time of its association with Cdc45 [2]–[5]. We therefore investigated the ratio of K36me1 and –me3 at replication origins and compared it to their time of activation, as reported in [46]. ARS609 lies within a gene-poor subtelomeric region where the average density of K36me3 differs significantly from the rest of the genome and was therefore excluded from the analysis [47]. A clear increase of K36me3/K36me1 ratio was observed for late replicating origins (Fig. 5A and Fig. S5). It is interesting to note that the major outlier ARS1524 does not advance its time of Cdc45 binding in the *Δrpd3* mutant (Fig. S2) and therefore may be subject to other regulatory mechanisms. Overall, a correlation factor of 0.76 was observed between the ratio of K36me3/K36me1 and the time of origin firing.

If this correlation is significant, then earlier firing origins should be either enriched in K36me1 or depleted in K36me3. We therefore determined K36me3 at origins at a genome-wide level. Two independent ChIP experiments using an antibody against K36me3 were hybridized against genomic DNA on yeast tiling arrays (Nimblegen). As expected, K36me3 was more prominent over coding regions when compared to intergenic regions (Fig. S6A, W = 1.98e11, P-value = 2.2e-16, using the Wilcoxon rank sum test with continuity correction). The location and timing data for known replication origins were extracted from oriDB (www.oridb.org and [48]). The average signal of K36me3 within the coordinates provided by the database was assigned to each replication origin. Replication origins were then clustered in 11 groups according to their time of firing and the distribution of K36me3 within each group was analysed. Replication origins that fired within six min of the activation of the first origin had significantly lower levels of K36me3 (Fig. 5B, group 1 and 2, W = 1142.5, P-value = 0.006). Moreover, excluding all subtelomeric origins within 30 kb of the chromosome end raises the mean distribution of K36me3 for later firing origins and further raises the significance of K36me3-depletion at early origins (W = 985.5, P-value = 0.003) (Fig. 5C). Similar results were obtained when using timing-data from [46] (data not shown). Because the resolution of ChIP is limited by the average fragment size of 500 bp of DNA, it was possible that the size of intergenic regions biased our results. If so, early origins would be expected to reside within longer intergenic regions. We were unable to identify significant differences in the length of intergenic regions containing group1 and 2 ARSs when compared to later firing ARSs (Fig. S6B, W = 1817, P-value = 0.66)

We conclude therefore that non-telomeric late origins are enriched in K36me3 compared to early firing origins, which show particularly low levels of this modification.

### K36me1 increases and K36me3 decreases during S-phase

If K36me1 and –me3 have opposite effects on Cdc45 binding to replication origins, they may have opposite dynamics during DNA replication. We therefore tested the possibility of transient changes in these two histone modifications during S-phase. Using lysate-samples from the experiment described in figure 2, we analysed the level of H3 K36me1 and –me3 by ChIP. The precipitated DNA was amplified by semiquantitative PCR and ARS-specific enrichment was normalized to the loading control and the input, which accounts for changing copy number between different chromosomal locations during S-phase (Fig. 5D and 5E). As histone deposition occurs immediately behind the replication fork, such normalization also accounts for differences due to nucleosome assembly [49]. In this time-course, Cdc45 association peaked at 28–36 min at ARS305 and at 44–52 min at ARS603 in WT cells (Fig. S3). We observed a 2.5–3 fold increase in K36me1 from 0 to 68 min for early ARS305 and 0 to 76 min for late ARS603 in the WT (Fig. 5E upper panels, full line). Similar results were obtained in the *Δrpd3* strain (Fig. 5E upper panels, dashed lines). This increase represents the net increase in K36me1 once the replication fork has passed, as normalization to the input hides changes due to nucleosome deposition. Omitting normalization to the input reveals that increases in K36me1 start concomitantly with the peak of Cdc45 association and occur earlier in the *Δrpd3* strain compared to the WT at the late ARS603 (Fig. S7). This may suggest a link between K36me1 and nucleosome assembly.

Analysis of K36me3 revealed a very different pattern. At the early ARS305 K36me3 decreased steadily by ∼2.5-fold between 0 and 44 min and a similar decrease was observed for late ARS603 between 36 and 52 min. Decreased K36me3 is observable even when the data are not normalized to increased copy number (Fig. S6). K36me3 levels in the *Δrpd3* strain were similar to WT at ARS305. At the late ARS603 this histone modification showed slightly lower levels in G1 and stayed at approximately the same level throughout the time of analysis in this experiment, indicating possible transient cell-cycle specific differences in K36me3 between the two strains. Similar data were obtained in two independent time-courses and at other replication origins (data not shown). We therefore conclude that K36me1 increases while K36me3 decreases upon Cdc45 association with replication origins.

### *EAF3* and *NTO1* act together to delay S-phase progression

**Figure 6:**
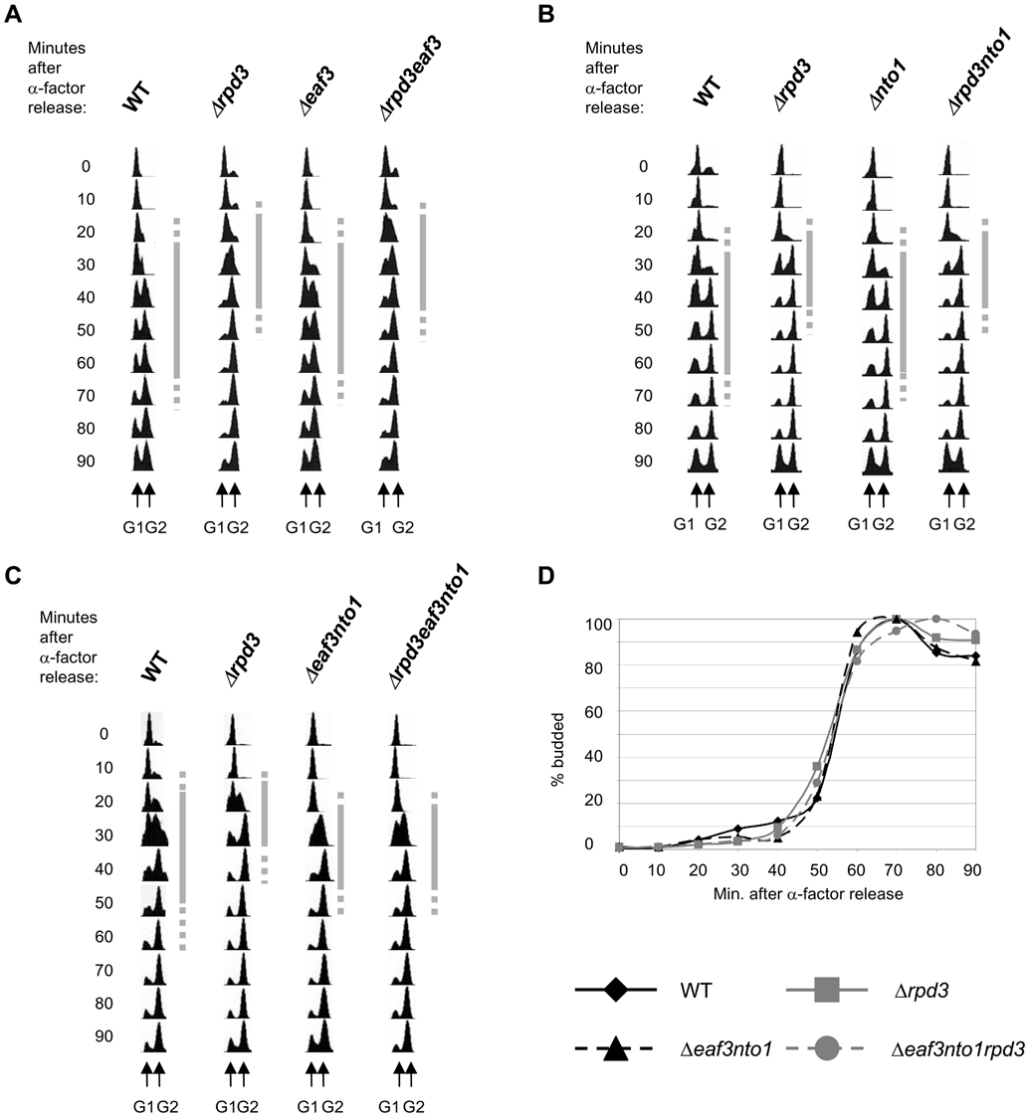
*EAF3* and *NTO1* act together to delay S-phase progression. (A) Strains MMY033 (WT), MVY51 (Δ*rpd3*), MVY54 (Δ*eaf3*) and MVY55 (Δ*eaf3*Δ*rpd3*) were arrested in G1 with α-factor and released into S-phase at 30°C. Samples were taken at indicated times and processed for FACS analysis. (B) as (A) with strains MMY001 (WT), MMY002 (Δ*rpd3*), MMY118 (Δ*nto1*) and MVY119 (Δ*rpd3*Δ*nto1*). (C) as (A) with strains MMY001 (WT), MMY002 (Δrpd3), MVY137 (Δ*eaf3*Δ*nto1*) and MVY138 (Δ*rpd3*Δ*eaf3*Δ*nto1*). Grey bars indicate the estimated length of S-phase. (D) Cell budding was assessed by microscopy. Complete lines indicate WT (black) and Δ*rpd3* (grey); broken lines indicate Δ*eaf3*Δ*nto1* (black) and Δ*rpd3*Δ*eaf3*Δ*nto1* (grey).

If K36me3 delays the association of Cdc45 with origins, deletion of proteins that bind this modification should result in a shortening of S-phase, similar to *Δrpd3*. Eaf3 and Nto1 are two factors that have been shown to bind H3 K36me3 [36]–[38], [42]. Eaf3 is a non essential subunit of the NuA4 HAT complex and part of the Rpd3(S) complex, while Nto1 is a subunit of the NuA3 HAT complex [44], [50], [51]. We therefore deleted each of these factors singly or in combination and analysed S-phase progression by FACS analysis. S-phase progression was not affected by the deletion of *EAF3* or *NTO1* alone, as the single mutants progressed through S-phase with similar kinetics to the WT (Fig. 6A and 6B). As expected, DNA replication occurred more rapidly in the *Δrpd3* strain and was not further accelerated by additional deletion of *EAF3* or *NTO1*. However, when both *EAF3* and *NTO1* were deleted S-phase was accelerated, similar to the *Δrpd3* strain. The triple mutant *Δeaf3Δnto1Δrpd3* was similar to the *Δeaf3Δnto1* double mutant, indicating that S-phase could not be further shortened by the deletion of *RPD3* (Fig. 6C). These data show that K36me3-binding proteins Eaf3 and Nto1 act redundantly to delay S-phase progression via a mechanism that is genetically dependent on *RPD3*.

Based on these results, we propose that methylation of H3 K36 plays an important role in determining the time of Cdc45 binding to replication origins and that the role of K36me1 and –me3 is opposing, with K36me1 advancing and K36me3 delaying this association.

## Discussion

Histone acetylation has been shown to positively regulate the time of Cdc45 association with replication origins and their subsequent time of firing. Recruitment of a HAT to a late replication origin advances its association with Cdc45 and its time of firing in yeast [4]. This function of histone acetylation has been confirmed by similar studies in human cells [27]. The role of histone acetylation is further confirmed by a direct interaction between the HATs and the Origin Recognition Complex (ORC) [52], [53]. Nevertheless, histone acetylation is unlikely to be the sole chromatin modification to regulate the activation of replication origins and similar to the regulation of gene transcription may act in concert with a multitude of other chromatin modifications. We show here that in *S.cerevisiae* methylation of H3 K36 takes part in this process and therefore represents a novel chromatin mark to regulate replication timing.

We first identified the H3 K36/37R and the *Δset2* mutation as suppressors of the shortened S-phase in the *Δrpd3* mutant. Increased histone acetylation in the *Δrpd3* mutant shortens S-phase by advancing the timing of Cdc45 binding to late replication origins and causing their earlier activation. Deletion of *SET2* delays Cdc45 binding to replication origins, suggesting a positive function of H3 K36 methylation in replication initiation. Furthermore, this delay cannot be reversed by deleting *RPD3*, implying that methylation of H3 K36 is necessary to allow earlier binding of Cdc45 to replication origins in conditions of histone hyperacetylation. The suppression of the shortened S-phase and the advanced Cdc45 binding in the *Δrpd3* mutant upon deletion of *SET2* likely reflect a concomitant change in the timing of origin firing, but requires absolute confirmation by a more direct experimental approach, such as 2-D gel analysis.

As histone acetylation at origins is not reduced in the absence of *SET2* and K36me only moderately increases in the *Δrpd3* strain, we conclude that these modifications must act together to facilitate the conversion from the pre-RC to the RC. In fact, complexes that bind to chromatin often contain more than one histone binding domain (reviewed in [54], [55]). Moreover, some replication factors associate in an interdependent manner [56], [57]. Interactions of multiple proteins with different histone modifications may therefore be part of the same assembly network.

The positive effect of Set2 on time of Cdc45 binding to replication origins was surprising, as K36me3 over transcribed coding regions recruits the Rpd3(S). Deletion of *SET2* was therefore expected to accelerate origin firing to some extent [36]–[38]. Moreover, *Δset2* suppresses the sensitivity to HU of an *spt16-11* mutant, supporting a negative role of this histone methyltransferase in DNA replication [58]. These apparently contradictory observations led us to consider K36me1 and K36me3 as separate signals that may have opposite functions in DNA replication. Indeed, the ratio of K36me3/K36me1 increased at most later firing origins analysed in this study. The simplest explanation for such a tendency would be that K36me1 helps to activate replication origins, but its effect can be counteracted by K36me3. The resulting prediction that early origins are depleted in K36me3 holds true at a genome-wide level. Later firing origins had a more widespread distribution of K36me3. Interestingly, the mean level of K36me3 of later firing origins further increased when those located within 30 kb from the telomere were excluded from the analysis. A genome-wide study of replication timing classified replication origins within approximately 35 kb from the telomere in a distinct group with an overall later activation time compared to the rest of the genome [6]. K36me3 may therefore affect only internal late origins, while subtelomeric origins may be delayed by other mechanisms, including low levels of K36me1 and histone acetylation.

Since the timing of replication origin firing, and Cdc45 binding to replication origins, has been shown to be dictated by the chromosomal environment [18], [19], it is possible that the effect of histone modifications on origin activation is purely context driven. If this was the case, then one would not expect histone modifications to change during origin activation. On the other hand, activation of replication origins could involve mechanisms that modify adjacent nucleosomes directly. In this scenario the local chromatin structure at any given origin would aid or resist these mechanisms. For K36me the latter is true. Indeed, analysis of K36me1 and K36me3 in synchronous cells undergoing DNA replication reveals an increase in K36me1 and a decrease in -me3 at replication origins at approximately the time of Cdc45 binding. K36me3 is not completely reduced to background levels, which may be due to the limitations of our analysis. Although cells are progressing synchronously through S-phase, the activation of an origin is a single event that occurs in only a subset of the entire population at any given time. The decrease of K36me3 raises the possibility that a histone demethylase is directly recruited and aids origin activation. Alternatively, changes in K36me3 and -me1 levels could be achieved through nucleosome disassembly and assembly at the initiating replication fork. While the exact mechanism demands further investigation, the switch in the level of K36me3 versus K36me1 at the time of Cdc45 association with origins suggests a direct involvement of these histone modifications in the initiation of DNA replication.

The low level of K36me3 at early firing ARSs and its decrease during origin activation both support an inhibitory function of this modification in origin firing. K36me3 signals for binding of Eaf3 and Nto1 to histone H3 [36]–[38], [42]. Deleting both of these proteins leads to a shortened S-phase, suggesting a negative role of these factors in DNA replication which may be mediated by H3 K36me3. The Eaf3 chromodomain protein is part of the Rpd3(S) complex and recruits the complex over coding regions via direct binding to methylated K36 [36]–[38], [59]. It is also a non-essential subunit of the NuA4 HAT complex [50], [51]. The PHD finger protein Nto1 is a subunit of the NuA3 HAT complex [44]. The association of NuA3 with chromatin is partially dependent on lysine 36 and Set2 [60]. While the inhibiting function of Eaf3 could be due to the recruitment of Rpd3(S), it is surprising that recruitment of a HAT complex would have a similar effect. A recent study proposes competition between Rpd3(S) and NuA4 [58]. NuA4 is the only essential HAT complex in *S.cerevisiae* and has been proposed to function in DNA replication [58], [61]. It is possible that all three complexes compete for their substrates. Deletion of both *EAF3* and *NTO1* may result in increased recruitment of NuA4 and so accelerate DNA replication.

Our data cannot entirely exclude the possibility that Set2 affects replication initiation indirectly. Assays that could rule out this possibility, such as targeting of Set2 to a single origin, are of difficult interpretation due to the crosstalk of H3 K36 methylation and histone acetylation. However, changes in gene expression in the *Δset2* strain are mediated by the Rpd3S complex [29]. If Set2 were to control replication origins indirectly, then deletion of *EAF3* alone should affect DNA replication in a similar manner to *Δset2.* Our data strongly argue against this possibility (Fig. 6). Furthermore, several observations support a direct involvement of H3 K36 methylation in regulating the timing of Cdc45 binding to replication origins. The similarity of FACS profiles between the H3 K36/37R mutant and the *Δset2* mutant argues for histones as the relevant substrate for the effect of Set2 on replication origins. Moreover, different replication origins are affected to a different extent by the deletion of either *SET2* (compare ARS409 and ARS1524 – WT and *Δset2* in Fig. S3) or *RPD3* (compare ARS603 and ARS1524 – WT and *Δrpd3* in Fig. S3). Such variability between single replication origins should not be observed, if deletion of *SET2* merely alters the expression of a replication factor. Finally, changes of H3 K36me1 and – me3 during DNA replication strongly argue that these histone modifications are part of this process and therefore changes in the local environment of replication origins are likely to affect the dynamics of Cdc45 binding directly.

**Figure 7:**
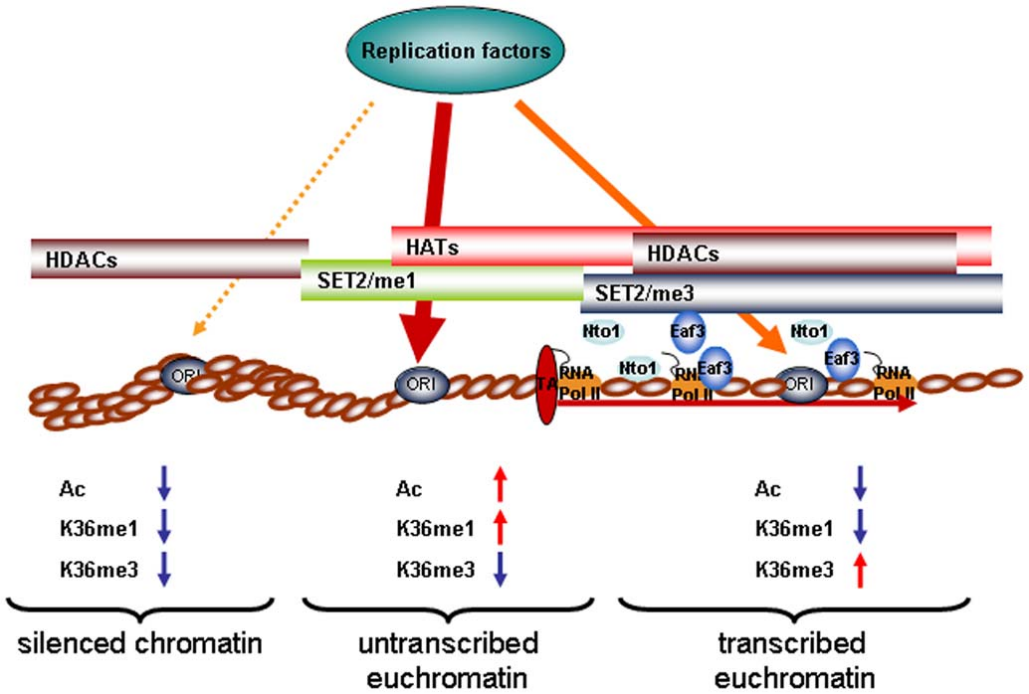
Model for action of histone modifications at replication origins. Replication factors have differential affinity for chromatin regions. Low affinity occurs at regions with low levels of histone acetylation and absence of K36me (silenced chromatin, left) and high levels of K36me3 (transcribed chromatin, right). High levels of K36me1 and histone acetylation cause high affinity for replication factors (center).

The participation of K36me in regulating the kinetics of Cdc45 binding to replication origins may have important implications in regulating the choice of origin usage. Potential preRCs are scattered along chromosomes. Some of these loci may reside within chromosomal regions that endanger the correct establishment of a topologically complex structure such as a functional RC. It would be advantageous to convert only those pre-RCs to RCs that lie within chromosomal regions posing the least problematic environment. Nevertheless, conversion of too few pre-RCs into RCs can result in chromosomal instability [62]. Differential timing of such conversions presents a solution to this dilemma by limiting this event to occur prevalently within the most favourable chromosomal environments, while ensuring formation of sufficient replicators. From this perspective, a pre-RC within a repressive environment will eventually be converted to a functional RC, but only if not prevented by a passing replication fork deriving from a nearby more favourable chromosomal environment [63], [64]. Histone modifications are the ideal candidates to inform the replication apparatus about the nature of the chromosomal environment. Both histone acetylation and K36me1 are depleted from heterochromatin. Higher order chromatin may present a topologically restrained environment for the initiation of a functional bidirectional replication fork. K36me3 marks chromosomal regions of ongoing transcription by RNA pol II [30], [31], [33], [34]. The dynamics of transcription and the resulting changes in DNA topology may interfere with the successful establishment of the RC (reviewed in [65]–[67]). In fact, origin usage is profoundly altered by transcription within euchromatic regions [22]–[24]. We therefore propose that methylation of H3 K36 is part of a signaling-network of histone modifications that informs the replication apparatus about its choice of origin usage by either favouring or inhibiting its interaction with chromatin (Fig. 7).

## Materials and Methods

### Yeast media and α factor arrest

Yeast cultures were grown at 30°C in rich medium (YPDA) unless otherwise stated. For α-factor arrest cells were grown to a density of 0.5 at OD_600_, incubated in fresh media containing 1 µg/ml α−factor for 2–3 hours and then released from arrest by incubation in fresh media containing 50 µg/ml pronase after washing twice with H_2_O.

### FACS analysis

FACS analysis was performed essentially as described previously, except that ProteinaseK treatment was omitted and cells were stained with 1 µM SYTOX Green (Molecular Probes) [68]. Entry into S-phase was estimated visually upon broadening of the G1 peak.

### Yeast Strains

Yeast strains in this study are isogenic to RMY200 [69] or YDS2 [70]. Gene disruptions were performed by one-step gene deletion [71]. A list of strains used in this study is shown in Table S1.

### Budding analysis

An equal volume of fixing solution (0.9% NaCl, 3.7% formaldehyde) was added to 500 µl aliquots of cells. 250–300 cells were counted and the percentage of budded cells calculated.

### Chromatin immunoprecipitation

50 ml of 1OD_600_ yeast cultures were formaldehyde crosslinked at room temperature for 15–20 min. ChIP was performed as described elsewhere [72], [73]. All antibodies used in this study were tested for specificity and titrated using appropriate mutant strains (data not shown). Immunoprecipitation of CDC45-FLAG3 using the αFLAG-M2 antibody (Sigma) was carried out as described in [4]. A list of antibodies used in this study is provided in Table S2.

### ChIP on CHIP

Immunoprecipitated and genomic DNA was amplified as described in [74]. Labelling, hybridization on whole genome tiling arrays for *S.cerevisiae* and analysis was performed by Nimblegen (www.nimblegen.com). The complete data-set is available in ArrayExpress with accession number E-TABM-497.

### Primer sequences

Primer sequences for all PCR fragments used in this study are available upon request.

## Supporting Information

Figure S1 Linearity of PCR reactions Different amounts (0.5–1–2–4 µl) of Cdc45-FLAG chromatin-immunoprecipitated DNA and of the corresponding Input-DNA were amplified by PCR to attest linearity of the reaction. The resulting gel (top) was vacuum-dried and analysed by Phosporimaging and the intensities reported in the graphical representations (lower part of the figure). 2 µl was chosen for amplification of Cdc45-FLAG and Input samples.
(0.13 MB TIF)

Figure S2 Set2p is necessary for accelerated DNA replication in Δrpd3 cells. Exponentially growing cells of strains MVY17 (WT), MVY31 (Δrpd3), MVY57 (Δset2) and MVY58 (Δrpd3Δset2) were arrested in G1 with α-factor and released into S-phase at 24°C. Samples were taken at indicated times and processed for FACS analysis. Grey bars indicate the estimated length of S-phase.
(0.10 MB TIF)

Figure S3 Set2p is necessary for advanced binding of cdc45 in WT and Δrpd3 cells. Exponentially growing cells of strains MVY17 (WT), MVY31 (Δrpd3), MVY57 (Δset2) and MVY58 (Δrpd3Δset2) were arrested in G1 with α-factor, released at 24°C into S-phase and samples were taken at indicated times. ChIP of Cdc45-3FLAG was performed with α-FLAG antibody and analysed by semiquantitative PCR using primers specific for the indicated ARS elements and a telomeric loading control (TEL). Graphical representation of Cdc45-3FLAG ChIP showing the relative intensity of ARS-specific fragments after normalization to the loading control and the input is presented. Complete lines indicate WT (black, diamonds) and Δrpd3 (grey, squares); broken lines indicate Δset2 (black, diamonds) and Δrpd3Δset2 (grey, squares).
(0.09 MB TIF)

Figure S4 Hyperacetylation at replication origins due to loss of Rpd3p is unaffected by the deletion of SET2. Alternative graphic representation of data presented in figure 3. The graph represents the average ratio (Δset2/WT or Δrpd3Δset2/Δrpd3) of histone acetylation of three independent experiments after normalization to input DNA and loading control. Error-bars refer to the standard deviation thereof.
(0.08 MB TIF)

Figure S5 Early origins are depleted in K36me3 compared to late firing origins. ChIP of MVY17 (WT) and MVY31 (Δrpd3) was performed with antibodies specific for H3 K36me1 and −me3. Analysis was by semiquantitative PCR using primers specific for the indicated ARS elements and a telomeric loading control (TEL). The relative intensity of ARS specific fragments after normalization to the loading control and the input is presented for WT (A) and Δrpd3 (B). The graphs represent the average of three independent experiments. Error-bars refer to the standard deviation. The K36 m3/m1 ratio for each origin is also presented (C).
(0.08 MB TIF)

Figure S6 The length of intergenic regions does not correlate with the time of origin firing. H3 K36me3 levels over coding regions were compared to H3 K36me3 levels over intergenic regions genomewide (A). The length of intergenic regions containing ARSs were taken from the Saccharomyces genome database (www.yeastgenome.org) and grouped for their time of replication as in Fig.6B (B). W- and P-values described in the text were calculated using the Wilcoxon rank sum test with continuity correction.
(0.08 MB TIF)

Figure S7 K36me1 increases and K36me3 decreases during S-phase. ChIP of MMY033 (WT) and MVY51 (Δrpd3) with antibodies specific for H3 K36me1 and −me3 was performed. Graphical representation of K36me1 (A) or K36me3 (B) ChIP showing the relative intensity of ARS-specific fragments after normalization to only the loading control is presented. Complete lines indicate WT and broken lines indicate Δrpd3.
(0.06 MB TIF)

Table S1 Strains used in this study
(0.05 MB DOC)

Table S2 Antibodies used in this study
(0.03 MB DOC)
