# Supplementary figures and images for: H3 K36 Methylation Helps Determine the Timing of Cdc45 Association with Replication Origins
Source: PLoS One. 2009 Jun 12;4(6):e5882. doi: 10.1371/journal.pone.0005882 (PMC2690658; doi:10.1371/journal.pone.0005882)

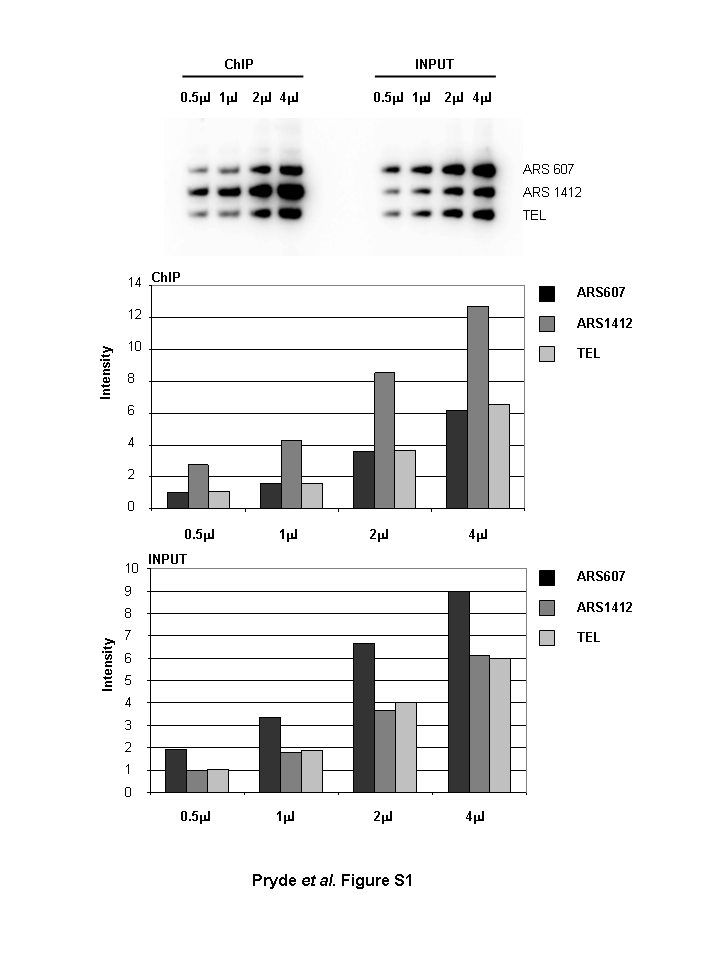

Supplement: Figure S1 — Linearity of PCR reactions Different amounts (0.5–1–2–4 µl) of Cdc45-FLAG chromatin-immunoprecipitated DNA and of the corresponding Input-DNA were amplified by PCR to attest linearity of the reaction. The resulting gel (top) was vacuum-dried and analysed by Phosporimaging and the intensities reported in the graphical representations (lower part of the figure). 2 µl was chosen for amplification of Cdc45-FLAG and Input samples. (0.13 MB TIF) [file pone.0005882.s001.tif]

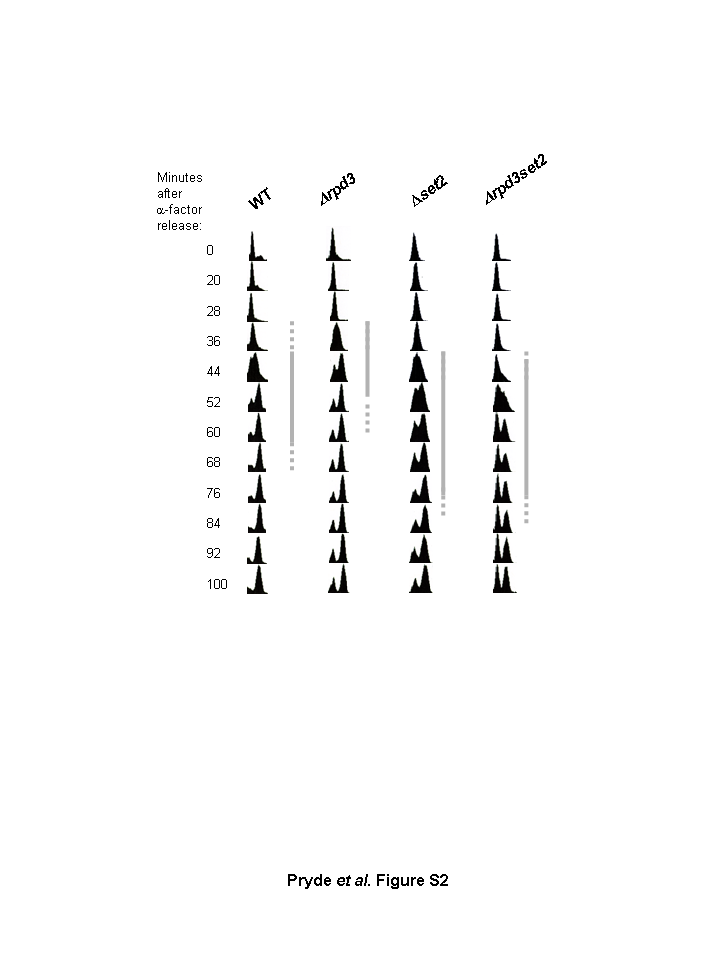

Supplement: Figure S2 — Set2p is necessary for accelerated DNA replication in Δrpd3 cells. Exponentially growing cells of strains MVY17 (WT), MVY31 (Δrpd3), MVY57 (Δset2) and MVY58 (Δrpd3Δset2) were arrested in G1 with α-factor and released into S-phase at 24°C. Samples were taken at indicated times and processed for FACS analysis. Grey bars indicate the estimated length of S-phase. (0.10 MB TIF) [file pone.0005882.s002.tif]

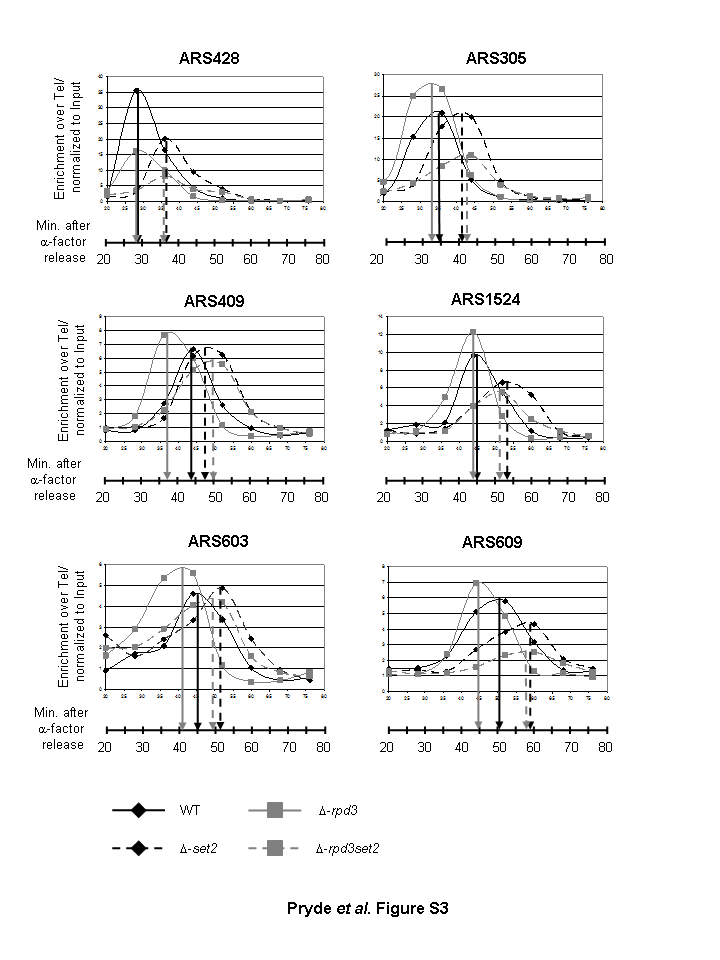

Supplement: Figure S3 — Set2p is necessary for advanced binding of cdc45 in WT and Δrpd3 cells. Exponentially growing cells of strains MVY17 (WT), MVY31 (Δrpd3), MVY57 (Δset2) and MVY58 (Δrpd3Δset2) were arrested in G1 with α-factor, released at 24°C into S-phase and samples were taken at indicated times. ChIP of Cdc45-3FLAG was performed with α-FLAG antibody and analysed by semiquantitative PCR using primers specific for the indicated ARS elements and a telomeric loading control (TEL). Graphical representation of Cdc45-3FLAG ChIP showing the relative intensity of ARS-specific fragments after normalization to the loading control and the input is presented. Complete lines indicate WT (black, diamonds) and Δrpd3 (grey, squares); broken lines indicate Δset2 (black, diamonds) and Δrpd3Δset2 (grey, squares). (0.09 MB TIF) [file pone.0005882.s003.tif]

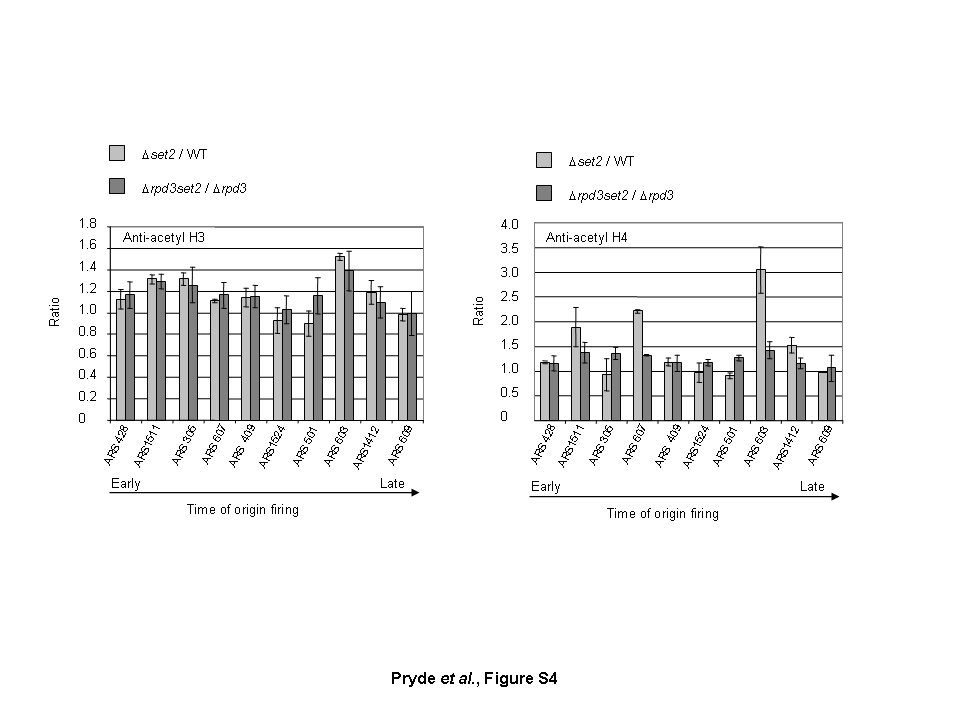

Supplement: Figure S4 — Hyperacetylation at replication origins due to loss of Rpd3p is unaffected by the deletion of SET2. Alternative graphic representation of data presented in figure 3. The graph represents the average ratio (Δset2/WT or Δrpd3Δset2/Δrpd3) of histone acetylation of three independent experiments after normalization to input DNA and loading control. Error-bars refer to the standard deviation thereof. (0.08 MB TIF) [file pone.0005882.s004.tif]

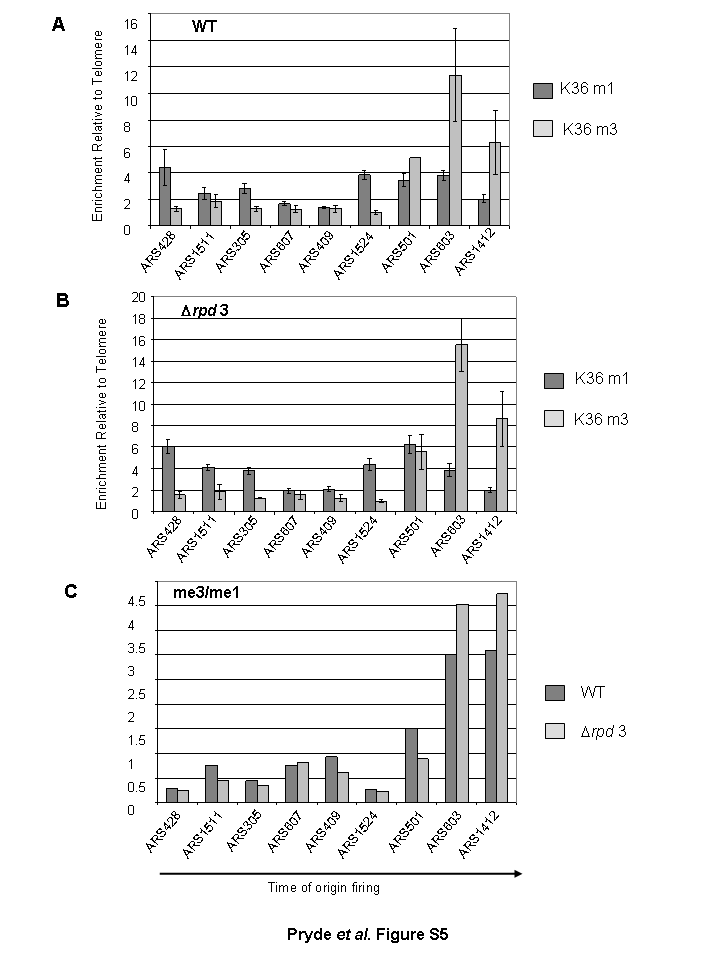

Supplement: Figure S5 — Early origins are depleted in K36me3 compared to late firing origins. ChIP of MVY17 (WT) and MVY31 (Δrpd3) was performed with antibodies specific for H3 K36me1 and −me3. Analysis was by semiquantitative PCR using primers specific for the indicated ARS elements and a telomeric loading control (TEL). The relative intensity of ARS specific fragments after normalization to the loading control and the input is presented for WT (A) and Δrpd3 (B). The graphs represent the average of three independent experiments. Error-bars refer to the standard deviation. The K36 m3/m1 ratio for each origin is also presented (C). (0.08 MB TIF) [file pone.0005882.s005.tif]

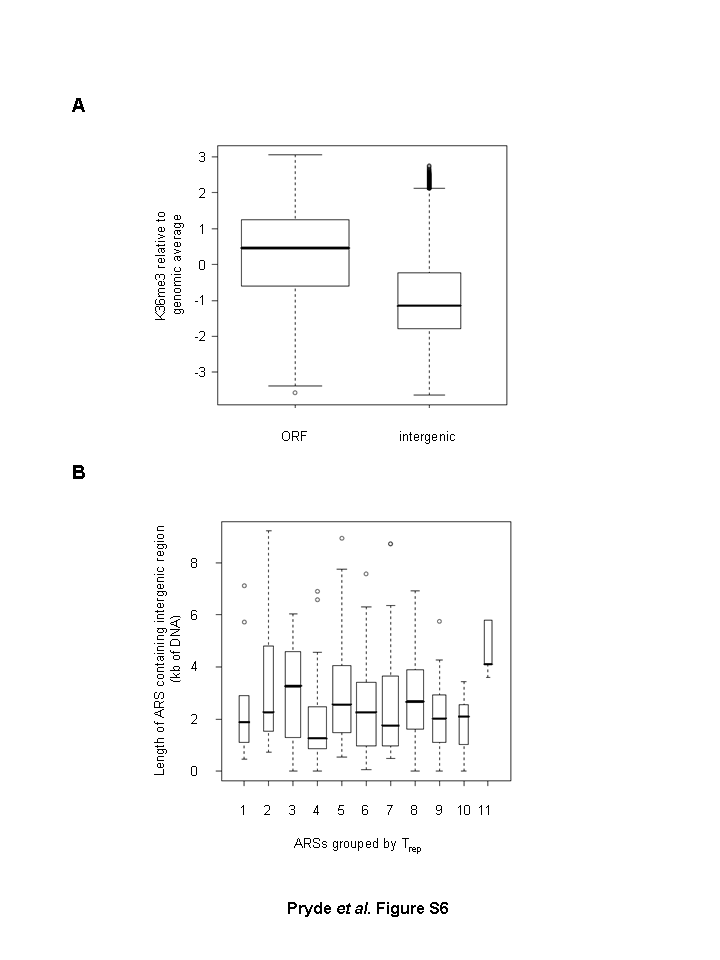

Supplement: Figure S6 — The length of intergenic regions does not correlate with the time of origin firing. H3 K36me3 levels over coding regions were compared to H3 K36me3 levels over intergenic regions genomewide (A). The length of intergenic regions containing ARSs were taken from the Saccharomyces genome database (www.yeastgenome.org) and grouped for their time of replication as in Fig.6B (B). W- and P-values described in the text were calculated using the Wilcoxon rank sum test with continuity correction. (0.08 MB TIF) [file pone.0005882.s006.tif]

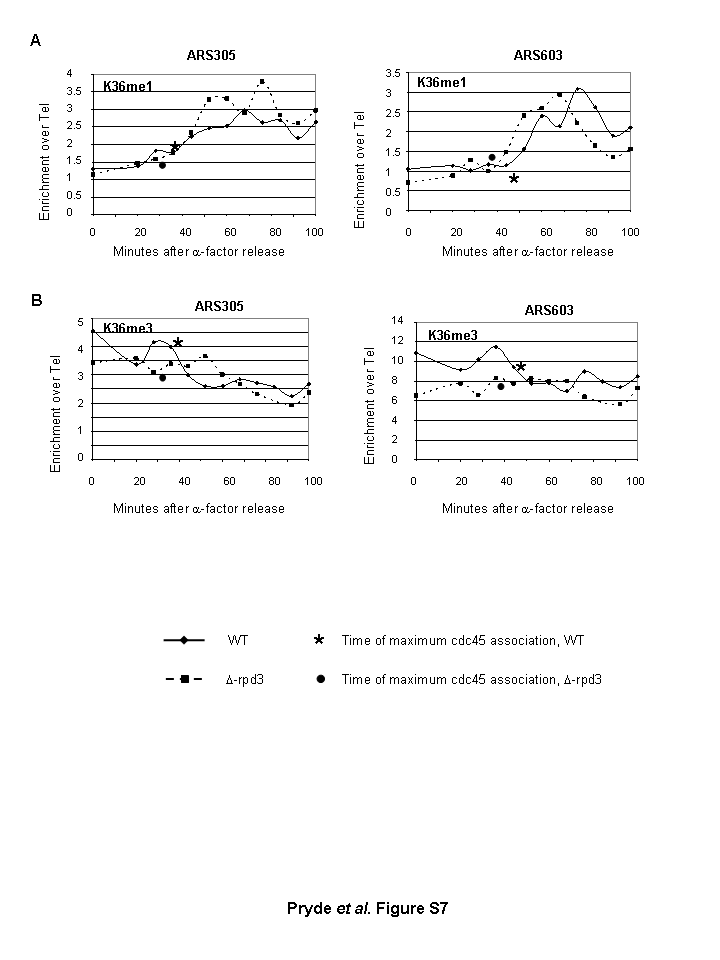

Supplement: Figure S7 — K36me1 increases and K36me3 decreases during S-phase. ChIP of MMY033 (WT) and MVY51 (Δrpd3) with antibodies specific for H3 K36me1 and −me3 was performed. Graphical representation of K36me1 (A) or K36me3 (B) ChIP showing the relative intensity of ARS-specific fragments after normalization to only the loading control is presented. Complete lines indicate WT and broken lines indicate Δrpd3. (0.06 MB TIF) [file pone.0005882.s007.tif]
