# Supplementary material for: H3 K36 Methylation Helps Determine the Timing of Cdc45 Association with Replication Origins
Source: PLoS One. 2009 Jun 12;4(6):e5882. doi: 10.1371/journal.pone.0005882 (PMC2690658; doi:10.1371/journal.pone.0005882)
Supplement: Table S1 — Strains used in this study (0.05 MB DOC) [file pone.0005882.s008.doc]

**Table S1**: Strains and plasmids used in this study

All strains are isogenic to RMY200 (Mann and Grunstein, 1992) or YDS2 (Laman *et al,* 1995).

| MMY001 | *MAT***a**, *ade2-1*, *can1-100*, *his3-Δ200,leu2-3,112*, *trp1*, *ura3-52, bar1::HIS3* | Vogelauer *et al*., 2002 |
| --- | --- | --- |
| MMY002 | *MAT***a**, *ade2-1*, *can1-100*, *his3-Δ200,leu2-3,112*, *trp1*, *ura3-52, rpd3::LEU2, bar1::HIS3* | Vogelauer *et al*., 2002 |
| MMY033 | *MAT***a**, *ade2-1*, *can1-100*, *his3-Δ200,leu2-3,112*, *trp1*, *ura3::LEU2, bar1::HIS3, Cdc45-*Flag*(kanMX)* | Vogelauer *et al*., 2002 |
| MVY16 | *MAT****a****, ade2-101(och), his3D200, lys2-801 (amb), trp1D901, ura3-52,bar1::hygB, hht1,hhf1::LEU2, hht2,hhf2::HIS3* plus pRM102 *(CEN4 ARS1 URA3* P(GAL10)-*HHT2* P(GAL1)-*HHF2)* | This study |
| MVY17 | *MAT****a****, ade2-101(och), his3D200, lys2-801 (amb), trp1D901, ura3-52, bar1::hygB, hht1,hhf1::LEU2, hht2,hhf2::HIS3* plus pRM200 *(CEN4 ARS1 TRP1 HHT2 HHF2)* | This study |
| MVY31 | *MAT****a****, ade2-101(och), his3D200, lys2-801 (amb), trp1D901, ura3-52,* *rpd3::KanMX6, bar1::hygB, hht1,hhf1::LEU2, hht2,hhf2::HIS3* plus pRM200 *(CEN4 ARS1 TRP1 HHT2 HHF2)* | This study |
| MVY32 | *MAT****a****, ade2-101(och), his3D200, lys2-801 (amb), trp1D901, ura3-52,bar1::hygB,* *rpd3::KanMX6, hht1,hhf1::LEU2, hht2,hhf2::HIS3* plus pRM102 *(CEN4 ARS1 URA3* P(GAL10)*-HHT2* P(GAL1)*-HHF2)* | This study |
| MVY34 | *MAT****a****, ade2-101(och), his3D200, lys2-801 (amb), trp1D901, ura3-52,bar1::hygB,* *rpd3::KanMX6, hht1,hhf1::LEU2, hht2,hhf2::HIS3* plus pMV3 | This study |
| MVY37 | *MAT****a****, ade2-101(och), his3D200, lys2-801 (amb), trp1D901, ura3-52,bar1::hygB, hht1,hhf1::LEU2, hht2,hhf2::HIS3* plus pMV3 | This study |
| MVY42 | *MAT***a**, *ade2-1*, *can1-100*, *his3-Δ200,leu2-3,112*, *trp1*, *ura3-52, set2::KanMX6, bar1::HIS3* | This study |
| MVY43 | *MAT***a**, *ade2-1*, *can1-100*, *his3-Δ200,leu2-3,112*, *trp1*, *ura3-52, set2::KanMX6, rpd3::LEU2, bar1::HIS3* | This study |
| MVY51 | *MAT***a**, *ade2-1*, *can1-100*, *his3-Δ200,leu2-3,112*, *trp1*, *ura3::LEU2,rpd3::TRP1, bar1::HIS3, Cdc45-*Flag*(kanMX)* | This study |
| MVY54 | *MAT***a**, *ade2-1*, *can1-100*, *his3-Δ200,leu2-3,112*, *trp1*, *ura3::LEU2, eaf3::HygB, bar1::HIS3, Cdc45-*Flag*(kanMX)* | This study |
| MVY55 | *MAT***a**, *ade2-1*, *can1-100*, *his3-Δ200,leu2-3,112*, *trp1*, *ura3::LEU2, eaf3::HygB, rpd3::TRP1, bar1::HIS3, Cdc45-*Flag*(kanMX)* | This study |
| MVY57 | *MAT***a**, *ade2-1*, *can1-100*, *his3-Δ200,leu2-3,112*, *trp1*, *ura3::LEU2, set2::HygB, bar1::HIS3, Cdc45-*Flag*(kanMX)* | This study |
| MVY58 | *MAT***a**, *ade2-1*, *can1-100*, *his3-Δ200,leu2-3,112*, *trp1*, *ura3::LEU2, set2::HygB, rpd3::TRP1, bar1::HIS3, Cdc45-*Flag*(kanMX)* | This study |
| MVY118 | *MAT***a**, *ade2-1*, *can1-100*, *his3-Δ200,leu2-3,112*, *trp1*, *ura3-52, nto1::HygB, bar1::HIS3* | This study |
| MVY119 | *MAT***a**, *ade2-1*, *can1-100*, *his3-Δ200,leu2-3,112*, *trp1*, *ura3-52, nto1::Hyg3, rpd3::LEU2, bar1::HIS3* | This study |
| MVY137 | *MAT***a**, *ade2-1*, *can1-100*, *his3-Δ200,leu2-3,112*, *trp1*, *ura3::LEU2, eaf3::HygB,nto1::clonNAT, bar1::HIS3, Cdc45-*Flag*(kanMX)* | This study |
| MVY138 | *MAT***a**, *ade2-1*, *can1-100*, *his3-Δ200,leu2-3,112*, *trp1*, *ura3::LEU2, eaf3::HygB,nto1::clonNAT, rpd3::TRP1, bar1::HIS3, Cdc45-*Flag*(kanMX)* | This study |
| pMV3 | As pRM200 except H3 K36/37R | This study |
