# Supplementary material for: H3 K36 Methylation Helps Determine the Timing of Cdc45 Association with Replication Origins
Source: PLoS One. 2009 Jun 12;4(6):e5882. doi: 10.1371/journal.pone.0005882 (PMC2690658; doi:10.1371/journal.pone.0005882)
Supplement: Table S2 — Antibodies used in this study (0.03 MB DOC) [file pone.0005882.s009.doc]

**Table S2:** Antibodies used in this study

| **Name** | **Antibody** | **Quantity used per 50μl of lysate** |
| --- | --- | --- |
| αH3ac | Upstate 06-599 Lot#1416550 | 4μl |
| αH4ac | Upstate 06-866 Lot#1349702 | 1μl |
| αH3K36me1 | Abcam ab9048 Lot#103329 | 2.5μl |
| αH3K36me3 | Abcam ab9050 Lot#395432 | 1μl |
| αFLAG-M2-sepharose beads | Sigma A2220 | 10-15μl |
